# Revisiting checkpoint inhibitors for myeloma: maintenance after stem cell transplant

**DOI:** 10.1172/JCI167346

**Published:** 2023-02-15

**Authors:** Erin W. Meermeier, P. Leif Bergsagel

**Affiliations:** Department of Internal Medicine, Division of Hematology/Oncology, Mayo Clinic Arizona, Scottsdale, Arizona, USA.

## Abstract

Multiple myeloma is a hematologic malignancy of plasma cells that manifests with bone marrow tumors causing lytic bone lesions. Autologous stem cell transplantation (ASCT) after high-dose chemotherapy and followed by prolonged maintenance therapy with lenalidomide (LEN) is an effective standard-of-care therapy for multiple myeloma. However, most patients ultimately relapse. Rational combination strategies that address immune dysfunction may prolong the durability of ASCT. In this issue of the *JCI*, Minnie and colleagues investigated the addition of a checkpoint inhibitor to LEN maintenance therapy after ASCT. They found that the immune checkpoint TIGIT was an optimal target in patient samples. In a syngeneic, immunocompetent multiple myeloma mouse model, blockade of TIGIT synergized with LEN maintenance by inducing immune protection, characterized in part by the expansion of polyfunctional T cells in the bone marrow. The treatment enhanced durable antimyeloma efficacy and has translatable implications.

## Immune escape

Multiple myeloma (MM) remains incurable for most patients, despite substantial improvements in treatment. Induction followed by high-dose melphalan and autologous stem cell transplantation (ASCT) is the frontline therapy for eligible, recently diagnosed MM patients (1). ASCT is consistently associated with improved progression-free survival. Induction, most commonly with three or four cycles of the proteasome inhibitor lenalidomide (LEN) and dexamethasone, quickly reduces tumor burden, at which time hematopoietic stem cells are mobilized from the bone marrow into the blood using granulocyte CSF. Patients are subsequently treated with high-dose melphalan, and then reinfused with their stem cell graft to repopulate the bone marrow with healthy cells. Immunomodulatory drugs, such as LEN, are commonly used as maintenance therapy after ASCT to reduce the risk of recurrence. Despite these optimized therapies, most patients ultimately relapse. Impediments to durability of ASCT activity include initial stage, high-risk genetic features, and the degree of response to initial chemotherapy, although these explanations remain controversial in the field (2, 3). Preclinical work previously published by Minnie and colleagues suggests immune dysfunction as an alternative contributing factor to early relapse after ASCT (4). Specifically, the authors discovered that disease progression is characterized by immune escape mechanisms in the bone marrow. Further optimization of maintenance is of clinical relevance to improve patient outcomes. Given that combination therapy is valuable in other settings for MM and that immune dysfunction is a well-characterized feature of MM, a timely question for the field is: could targeted immunotherapy be a safe and optimal addition to post-ASCT maintenance?

## Immune checkpoint inhibitors

In this issue of the *JCI*, Minnie and colleagues preclinically investigated the use of a TIGIT immune checkpoint inhibitor to increase the durability of ASCT (5). To examine this critical matter, they leveraged patient graft samples, an MM mouse model, their established preclinical ASCT protocol, and a TIGIT-targeting antibody with murine reactivity. This strategy builds upon previous reports from this group regarding MM escape due to T cell exhaustion (4). Another study (6) indicates that TIGIT blockade restores CD8^+^ T cell immunity against MM. In the current study, Minnie and coauthors evaluated TIGIT blockade in the context of preventing T cell exhaustion under current post-ASCT maintenance (LEN) and provided mechanistic underpinnings of the synergistic efficacy observed in their model (5).

The authors first evaluated immune checkpoint expression on T cells in patients’ ASCT graft samples. They found that the immune checkpoint TIGIT was selectively expressed on CD8^+^ T cells in peripheral stem cell grafts from patients with MM, but expression of other checkpoints, such as PD-1, remained low. They also distinguished between immune checkpoint expression by senescent T cells, which are common in individuals over 50 years old, and nonsenescent T cells. TIGIT was upregulated in nonsenescent T cells in patients with MM. Importantly, these data were in comparison to grafts from healthy individuals and provided key credentials for pursuing TIGIT as a specific target in MM (5).

Checkpoint inhibitors are a revolutionary pillar of modern cancer therapy used to treat many types of malignancies and remain an expanding area of clinical development. However, their use in treating MM has not been successful so far. In clinical trials (KEYNOTE trials 183 and 185), the combination of immunomodulatory imide drugs (IMiDs) with PD-1 inhibition, pembrolizumab in combination with pomalidomide for relapsed or refractory MM, and pembrolizumab in combination with LEN for newly diagnosed MM patients resulted in decreased overall survival, which halted the trials (7, 8). The mechanism behind this safety concern remains unresolved but warrants caution for future clinical trials utilizing IMiDs in combination with immune checkpoint inhibitors in MM. We can speculate that TIGIT blockade may provide a safer alternative due to preclinical mouse studies showing that *Tigit*-knockout mice do not have significantly compromised immune homeostasis, as opposed to *Pdcd1-* or *Ctla4*-knockout mice (9).

The authors ultimately tested the antimyeloma activity of TIGIT blockade in combination with LEN using a mouse model of SCT. Their underlying model utilized mice genetically modified to be LEN sensitive that were engrafted with a murine myeloma cell line. One caveat of the model involved the difference in LEN sensitivity between the host and tumor; only the host, but not the tumor, was sensitive to LEN. Mice received total body irradiation to mimic the effects of high-dose melphalan, followed by transplantation of an optimized dose of T cells derived from myeloma-bearing mice. Then, recipient mice were immediately treated with an Fc-intact antibody targeting murine TIGIT followed by LEN. Notably, the combination of anti-TIGIT with LEN in mice was safe and synergistically improved survival (5). We can speculate that in patients, where the tumor is likely sensitive to IMiDs, there may be additional synergism with TIGIT blockade and LEN or next-generation IMiDs.

This group previously established the immune subset primarily activated by TIGIT inhibition as CD8^+^ T cells (4, 6, 10). In the current study, Minnie and coauthors went on to dissect the synergistic response by depleting T cells or a subset of T cells, CD8^+^ T cells, from the donor graft. Both depletion strategies completely abrogated the synergistic activity (5). This finding demonstrates the importance of adoptively transferred T cells for antimyeloma activity in this setting. Moreover, the mice that survived long term after ASCT also displayed functional immune protection in a rechallenge experiment.

## Combination treatment

**Figure 1:**
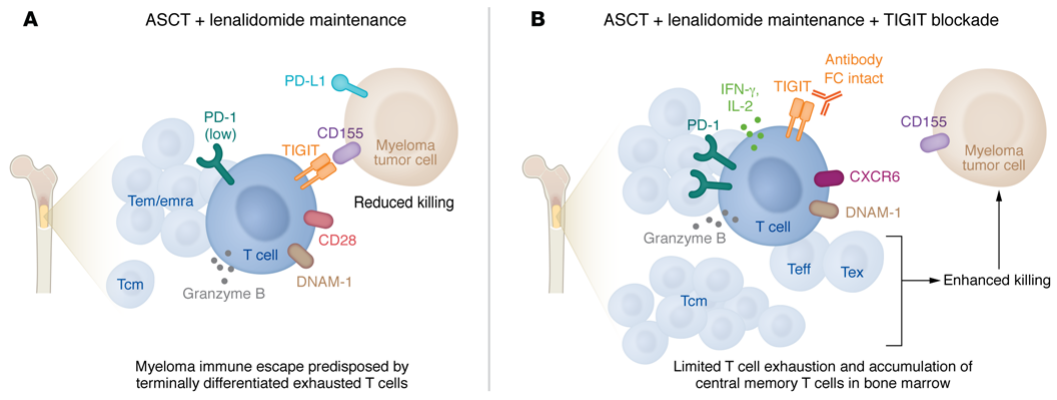
TIGIT blockade synergizes with LEN maintenance to reduce immune escape in MM. (**A**) The current standard of care for MM following ASCT involves maintenance with LEN treatment. However, most patients eventually relapse, and preclinical work suggests that immune dysfunction is a contributing factor to early relapse after ASCT. The study by Minnie et al. (5) discovered elevated levels of T cell exhaustion in mobilized peripheral blood stem cell grafts from patients with MM compared with healthy patients. Moreover, MM tumor cells may evade the immune system, in part, due to expression of the immune checkpoint TIGIT on effector memory (Tem) T cells and terminally differentiated effector memory T cells (Temra) in the bone marrow. (**B**) Antibodies that block TIGIT synergize with LEN maintenance by expanding polyfunctional T cells, including central memory T cells (Tcm), effector T cells (Teff), and exhausted T cells (Tex), in the bone marrow. Combination treatment in mice that includes TIGIT blockade with LEN maintenance limits T cell exhaustion, increases functional T cell accumulation in the bone marrow, and enhances durable anti-myeloma efficacy.

These results laid a foundation for exploring the mechanistic underpinnings of this combination synergy. The authors first examined changes in CD8^+^ T cell states using single-cell RNA-seq of bone marrow harvested from mice one month after ASCT. The combination treatment expanded populations of highly functional effector cells, characterized by production of key antitumor mediators like IFN-γ, IL-2, granzyme B, Fas ligand, and perforin (Figure 1). CD8^+^ T cells from mice exposed to TIGIT blockade alone or in combination with LEN also increased expression of DNAM-1, a receptor that competes for ligand with TIGIT, but imparts stimulatory activity instead of inhibitory effects. LEN alone or in combination with TIGIT blockade upregulated expression of IL-7R in memory T cells, which is important for memory homeostasis. Interestingly, TCR clonality analysis revealed the presence of hyperexpanded T cell clones within the CD8^+^ effector T cell subset that were also shared across exhausted T cell subsets. This phenomenon was not exclusive to the combination treatment, but found with single-agent treatment as well. The authors speculated that these T cell clones may be enriched for myeloma-specific T cells (5). Of note, clonal expansion of cytotoxic T cells is a clinically relevant phenotype observed in MM patients with exceptional responses after ASCT (11).

Minnie et al. additionally profiled T cells across the treatment groups at a later time point after contraction of the effector T cell response. They analyzed bone marrow–derived T cells using high-parameter flow cytometry. Most strikingly, TIGIT blockade plus LEN reduced exhausted T cells, and this combination was efficient at expanding the frequency of central memory CD8^+^ T cells. Surprisingly, these phenotypic changes were not observed in the peripheral blood (5). This specificity to bone marrow is a clinically relevant finding that highlights the importance of analyzing immune parameters of bone marrow samples during clinical evaluation in patients.

Further clinical investigations will be required to determine whether immunotherapy, including TIGIT checkpoint inhibitors, can be safely incorporated into maintenance treatments for MM to prolong progression-free survival and ultimately overall survival.

## Version 1 02/15/2023

Electronic publication
